# Powering Up Plant Genetic Research with Genomic Data 2.0

**DOI:** 10.3390/ijms26094052

**Published:** 2025-04-25

**Authors:** Man-Wah Li, Sachiko Isobe, Hon-Ming Lam

**Affiliations:** 1 Department of Molecular, Cellular and Developmental Biology, Yale University, New Haven, CT 06510, USA; 2 Lab of Horticultural Science, Graduate School of Agricultural and Life Sciences, The University of Tokyo, Tokyo 113-8654, Japan; sisobe@g.ecc.u-tokyo.ac.jp; 3 Centre for Soybean Research of the State Key Laboratory of Agrobiotechnology and School of Life Sciences, The Chinese University of Hong Kong, Hong Kong, China

Historically, plant genomic research has lagged behind mammalian studies, primarily due to the complexity of plant genomes and limited research resources. However, recent advances in high-throughput and long-read sequencing technologies have democratized access to high-quality genomic data [1], enabling efficient sequencing of extensive plant populations [2]. This technological leap has been complemented by remarkable progress in epigenomics, transcriptomics, proteomics, metabolomics, and phenomics, collectively providing a comprehensive understanding of plant systems.

Despite the vast genomic data now available in public repositories, translating this information into actionable insights remains a significant challenge. Each plant genome comprises vast sequences, often spanning hundreds of millions to trillions of base pairs, with tens of thousands of genes and numerous non-coding elements. To date, only a fraction of these genomic components has been functionally characterized, even in model organisms like *Arabidopsis thaliana*. This highlights the importance of focused research to annotate and elucidate the roles of these genetic elements.

Following the success of its first volume, this Special Issue, “Power Up Plant Genetic Research with Genomic Data 2.0”, brings together 16 articles showcasing diverse advancements in plant genomics and genetics, emphasizing the integration of genomic data to address complex challenges in plant science. Below is an overview of the contributions included in this issue.

High-quality genome assemblies are foundational to modern genomic research. As of 2024, approximately 1500 plant species have had their genomes sequenced and assembled, compared to about 6000 animal species [3]. Typically, genomes from model plants, economically important crops, and their wild relatives receive priority. In this issue, Radosavljević et al. present a chromosome-scale draft genome assembly of *Chouardia litardierei*, a species notable for its ecological plasticity (contribution 1). Furthermore, by resequencing genomes from meadow and mountainous ecotypes alongside a seashore ecotype, the authors highlight genomic diversity linked to ecological adaptation.

Well-assembled genomes enable gene discovery via both forward and reverse genetics. James et al. conducted a genome-wide association study (GWAS), narrowing down genomic loci and candidate genes associated with rice performance under low-phosphorus conditions in acidic soils (contribution 2). Additionally, Cheng et al., Zhou et al., and Yang et al. report the identification of the YTH gene family in the tea-oil tree (*Camellia chekiangoleosa*), the sulfate transporter gene family in soybean (*Glycine max*), and the APETALA2/Ethylene-responsive factor transcription factor family across seven *Solanaceae* species, respectively (contributions 3–5). To experimentally validate gene functions, Zhou et al. overexpressed the soybean sulfate transporter gene *GmSULTR3;1a* in yeast and soybean hairy roots, demonstrating enhanced salt tolerance and increased sulfur-containing compound accumulation (contribution 5). Yang et al. functionally characterized two locally duplicated *AP2* genes, *Capana01g004446* and *Capana01g004447*, from pepper (*Capsicum annuum*), confirming their roles in flowering regulation through gene silencing in pepper and ectopic expression in *Arabidopsis* (contribution 4).

Assembling chloroplast genomes represents a less resource-intensive alternative to nuclear genome sequencing. Due to their smaller size, uniparental inheritance, and lower mutation rates, chloroplast genomes are particularly suitable for evolutionary and phylogenetic studies [4]. This Special Issue includes an article utilizing chloroplast genomes to clarify phylogenetic relationships among eight *Lirianthe* Spach species (contribution 6).

Non-coding elements, such as promoters, microRNAs, and transposable elements, constitute significant portions of plant genomes, yet our understanding of these genomic “dark matter” components remains limited [5,6]. Several studies in this issue explore these elements: Sharif et al. identified and characterized the highly tissue-specific promoter of Naringenin 8-dimethylallyltransferase 2 (*AhN8DT-2*) from peanuts (*Arachis hypogaea*), demonstrating potential for tissue-specific genetic engineering (contribution 7). Yang et al. isolated and characterized a stress-responsive microRNA, miR2871b, from wild rice (*Oryza rufipogon*), revealing its detrimental effects under cold and salt-stress conditions (contribution 8). Additionally, Rao et al. examined integrated banana streak virus (BSV) sequences within *Musa* genomes, highlighting their distribution and coevolution with the host genome (contribution 9).

This Special Issue also includes several functional genomic studies. Wang et al. conducted a transcriptomic analysis of *Sorghum bicolor* L. under saline–alkaline stress (contribution 10). Sun et al. performed transcriptome-associated metabolomic profiling of Chinese bayberry (*Myrica rubra*) across three fruit developmental stages (contribution 11). Li et al. carried out a time-course transcriptomic comparison between grapevine cultivars with differing resistance to white rot (contribution 12). Qi et al. compared transcriptomes and coding sequences among *Arabidopsis thaliana*, *Arabidopsis lyrata*, and *Capsella rubella*, investigating how evolutionary divergence in gene expression and coding sequences contributes to functional differences among closely related species (contribution 13).

For genetic engineering and method development, Tesfaye et al. successfully enhanced erucic acid and wax ester content in *Brassica carinata* through genetic engineering, underscoring its potential as a source of long-chain fatty acids for industrial and biodiesel applications (contribution 14). Sidireddi et al. developed a specific loop-mediated isothermal amplification (LAMP) assay targeting a mild strain of *Xanthomonas citri* pv. *citri* (*Xcc* A^w^), facilitating efficient detection and management of citrus canker disease (contribution 15).

Finally, Gasparis et al. contributed an extensive review on the genetic control of grain size in important cereals such as rice, wheat, and barley, summarizing current understandings and highlighting future research directions (contribution 16).

The diverse contributions featured in this Special Issue highlight the remarkable progress in plant genomic research. We encourage researchers to build upon these discoveries and continue to power up plant genomic research.

## References

[1] Warburton P.E., Sebra R.P. (2023). Long-Read DNA Sequencing: Recent Advances and Remaining Challenges. Annu. Rev. Genom. Hum. Genet..

[2] Pucker B., Irisarri I., de Vries J., Xu B. (2022). Plant genome sequence assembly in the era of long reads: Progress, challenges and future directions. Quant. Plant Biol..

[3] Bernal-Gallardo J.J., de Folter S. (2024). Plant genome information facilitates plant functional genomics. Planta.

[4] Daniell H., Lin C.-S., Yu M., Chang W.-J. (2016). Chloroplast genomes: Diversity, evolution, and applications in genetic engineering. Genome Biol..

[5] Chi K.R. (2016). The dark side of the human genome. Nature.

[6] Jiang J. (2015). The ‘dark matter’ in the plant genomes: Non-coding and unannotated DNA sequences associated with open chromatin. Curr. Opin. Plant Biol..

